# Effect of Linker Elongation on the VGSC Affinity and Anticonvulsant Activity among 4-Alkyl-5-aryl-1,2,4-triazole-3-thione Derivatives

**DOI:** 10.3390/molecules28135287

**Published:** 2023-07-07

**Authors:** Kinga Paruch, Barbara Kaproń, Jarogniew J. Łuszczki, Agata Paneth, Tomasz Plech

**Affiliations:** 1 Department of Pharmacology, Faculty of Health Sciences, Medical University of Lublin, Radziwiłłowska 11, 20-080 Lublin, Poland; kinga.paruch@umlub.pl; 2 Department of Clinical Genetics, Faculty of Medicine, Medical University of Lublin, Radziwiłłowska 11, 20-080 Lublin, Poland; barbara.kapron@umlub.pl; 3 Department of Occupational Medicine, Faculty of Medical Sciences, Medical University of Lublin, Jaczewskiego 8B, 20-090 Lublin, Poland; jarogniew.luszczki@umlub.pl; 4 Department of Organic Chemistry, Faculty of Pharmacy, Medical University of Lublin, Chodźki 4A, 20-059 Lublin, Poland; agata.paneth@umlub.pl

**Keywords:** epilepsy, molecular modeling, 1,2,4-triazole-3-thiones, anticonvulsant activity, MES test, human VGSCs

## Abstract

The main aim of the current project was to investigate the effect of the linker size in 4-alkyl-5-aryl-1,2,4-triazole-3-thione derivatives, known as a group of antiepileptic drug candidates, on their affinity towards voltage-gated sodium channels (VGSCs). The rationale of the study was based both on the SAR observations and docking simulations of the interactions between the designed ligands and the binding site of human VGSC. HYDE docking scores, which describe hydrogen bonding, desolvation, and hydrophobic effects, obtained for 5-[(3-chlorophenyl)ethyl]-4-butyl/hexyl-1,2,4-triazole-3-thiones, justified their beneficial sodium channel blocking activity. The results of docking simulations were verified using a radioligand binding assay with [^3^H]batrachotoxin. Unexpectedly, although the investigated triazole-based compounds acted as VGSC ligands, their affinities were lower than those of the respective analogs containing shorter alkyl linkers. Since numerous sodium channel blockers are recognized as antiepileptic agents, the obtained 1,2,4-triazole derivatives were examined for antiepileptic potential using an experimental model of tonic–clonic seizures in mice. Median effective doses (ED_50_) of the compounds examined in MES test reached 96.6 ± 14.8 mg/kg, while their median toxic doses (TD_50_), obtained in the rotarod test, were even as high as 710.5 ± 47.4 mg/kg.

## 1. Introduction

The World Health Organization defines epilepsy as a chronic non-infectious disease of the brain. It is estimated that it affects almost 50 million people worldwide at every age group [1]. The hallmark of this disease is recurrent seizures, which are short episodes of involuntary movements that can involve the whole body or part of it. Treatment of epilepsy is difficult, primarily due to the fact that a large proportion of patients suffer from drug-resistant forms of the disease. In most patients, the currently available diagnostic methods fail to determine the cause of epilepsy which makes treatment even more difficult [2]. Depending on the age group, the etiology of epilepsy is different, so its treatment should also be different [3]. The most common way to manage epilepsy is pharmacological treatment. More than 30 antiepileptic drugs are currently available. Antiepileptic drugs can reduce the number of epileptic seizures, their intensity, and finally, effectively prevent them. Treatment starts with low doses of one drug and then its dose is increased. However, seizure control is sometimes possible only by combining several different antiepileptic drugs (AEDs) [4]. The new generations of AEDs currently include tiagabine, vigabatrin, lamotrigine, zonisamide, felbamate, oxacarbazine, topiramate, and gabapentine. Newer antiepileptic drugs have a better pharmacokinetic profile and better patient tolerance, but their effectiveness is not significantly higher than that of basic drugs. Therefore, there is still about 30% of patients with drug-resistant seizures who are waiting for effective therapeutic help [5,6]. Another problem that neurologists have to face is a number of side effects that are shown by AEDs available on the market. The most common side effects include fatigue and drowsiness, but these are not symptoms that threaten the patient’s health. On the other hand, some AEDs have hepatotoxic and teratogenic effects, which already threaten the health and life of patients [7,8]. When pharmacological treatment is ineffective in the treatment of epilepsy, one of the methods of non-pharmacological therapy is considered. These include the ketogenic diet, vagus nerve stimulation, and surgical treatment [9].

**Figure 1:**
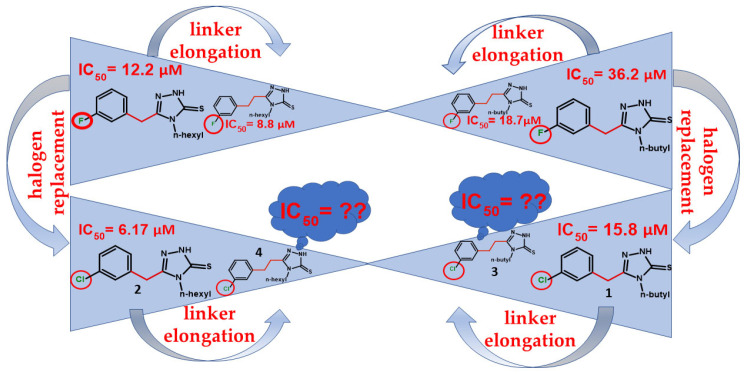
Rationale of the current studies (the numbering of compounds **1**–**4** is consistent throughout whole manuscript). The IC_50_ values were obtained from [15].

According to the literature, compounds possessing 1,2,4-triazole ring in their structures are characterized by high antiepileptic potential [10,11,12,13,14]. Among them, 4-alkyl-5-aryl-1,2,4-triazole-3-thione derivatives express anticonvulsant activity both against tonic–clonic seizures and in an animal model of drug-resistant epilepsy [15,16,17]. Mechanistic studies revealed that these compounds could not affect the GABAergic neurotransmission, neither directly nor allosterically; however, they acted as voltage-gated sodium channel ligands in a low micromolar range [18]. Both affinity towards VGSC and anticonvulsant potency of 4-alkyl-5-aryl-1,2,4-triazole-3-thione derivatives were most beneficial when the alkyl group was unbranched and had 4 to 7 carbon atoms [19]. Additionally, their pharmacological efficacy increased when short aliphatic linkers were applied to connect the 1,2,4-triazole-3-thione ring and an aryl moiety at its C5 position [11,15]. As proof, the introduction and subsequent elongation of the aliphatic linker, from –CH2– to –CH2–CH2–, among the respective 3-fluorophenyl derivatives, resulted in an improvement of affinity towards VGSCs from 190 to 36.2 to 18.7 µM, while the affinities decreased from 190 µM to 36.2 µM to 18.7 µM for the 4-butyl derivatives. Similarly, for the 4-hexyl derivatives, the affinities decreased from 35 µM to 12.2 µM to 8.8 µM of 1,2,4-triazole-3-thione (Figure 1) [15].

Similarly, the beneficial effect of the linker presence was observed in the case of 3-chlorophenyl derivatives. Thus, 5-(3-chlorobenzyl)-4-hexyl-1,2,4-triazole-3-thione blocked the VGSCs with IC_50_ of 6.17 µM [19]. Although further elongation of the methylene linker should, by analogy to the above-mentioned fluorophenyl derivatives, result in even more potent affinity against VGSCs, the respective 5-[(3-chlorophenyl)ethyl]-4-alkyl-1,2,4-triazole-3-thiones have never been obtained so far. Therefore, our current studies aimed at investigating the effect of 5-[(3-chlorophenyl)ethyl]-4-butyl/hexyl-1,2,4-triazole-3-thiones on VGSCs using both in silico docking simulations and, subsequently, the radioligand binding assay. Docking experiments were based on the previously validated model of human VGSC (pdb id: 5EK0), in which the in silico obtained free binding energies corresponded to the experimentally evaluated affinity of the respective 1,2,4-triazole derivatives [19]. Additionally, taking into account that the sodium channel ligands are broadly recognized as antiepileptic agents, the obtained 1,2,4-triazole derivatives were examined for their anticonvulsant effect using in the vivo model of tonic–clonic seizures, while the possible neurotoxic effects were evaluated in the rotarod test in mice.

There are 9 subtypes of the Nav channels, characterized by tissue-specific distribution, and associated with different diseases. However, these channels share a high degree of amino acid sequence similarity. Therefore, sodium channel blockers, although treat a range of cardiovascular and neurological disorders, usually lack subtype selectivity due to conservation of the central-pore drug binding site [20,21]. Considering the high similarity between different Nav subtypes and the limited set of proteins that can be used for docking, we decided to use the well-described Nav 1.7 channel protein. Mutations in the Nav 1.7 channel are mainly associated with pain disorders; however, current research shows some correlations between epilepsy and this channel, especially when it comes to febrile seizures and Dravet’s syndrome in humans [22,23,24]. It can be suspected that Nav 1.7 mutations cause hyperexcitability of sensory neurons and hypoexcitability of sympathetic neurons. These opposing electrical properties may be the basis for explaining how the same gene previously associated with peripheral pain may be associated with the epilepsy phenotype [23]. Studies conducted on a group of 21 people showed that SCN9A mutations cause febrile seizures, and another study conducted on mice showed that individuals with the SCN9A—N641Y mutation are more susceptible to tonic–clonic seizures caused by electric shock [24]. Moreover, there are reports that confirm the occurrence of mutations within Nav 1.7 in patients suffering from generalized epilepsy, temporal lobe epilepsy, and febrile seizures.

## 2. Results and Discussion

### 2.1. Molecular Docking

**Figure 2:**
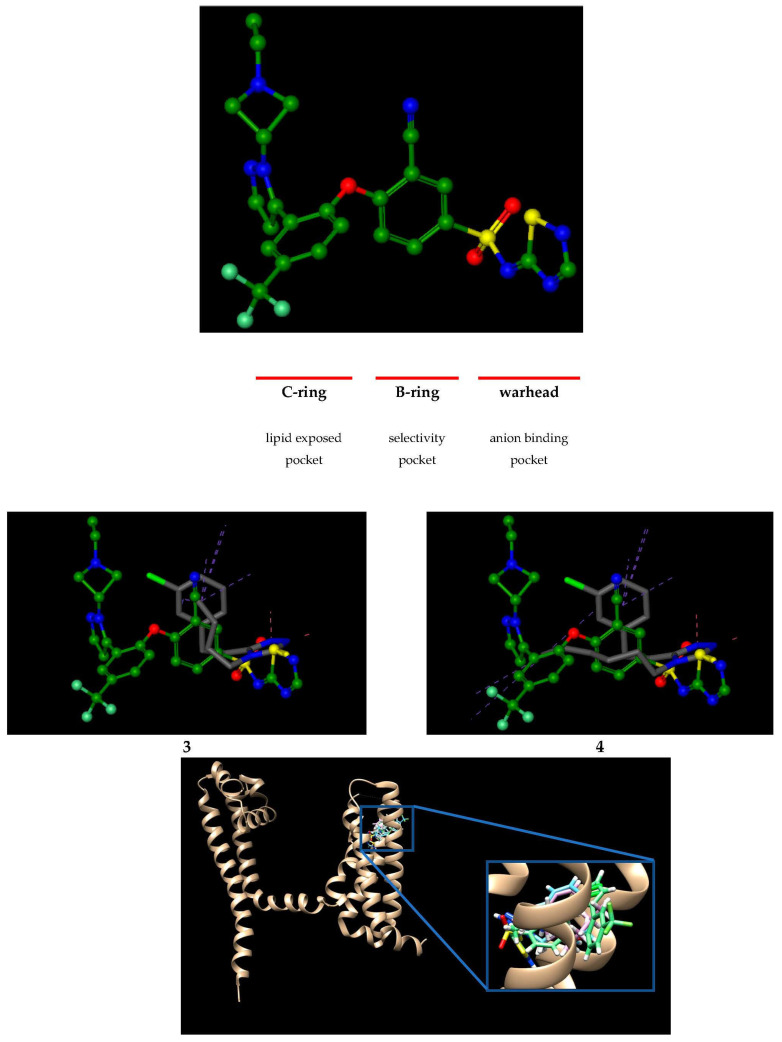
The best binding poses of the *s*-triazoles **3** and **4** overlaid with the native antagonist GX-936 at the human Nav1.7-VSD4-NavAb interface, along with their orientation at the chain D of the protein (pdb id 5EK0). For details see Figure 3 and Table 1, respectively.

In 2015 [25], the crystal structure of a novel Nav1.7 receptor site in a complex with the three-building-block sulfonamide GX-936 (Figure 2) was published which resolved the structural basis of Nav1.7 inhibition by an isoform-selective small molecule antagonist GX-936. The GX-936 binds to the activated state of the voltage-sensor domain IV (VSD4), where its anionic aryl sulfonamide warhead binds directly to the fourth arginine gating charge on the S4 helix (the R4 gating charge, Arg1608) through a bidentate salt bridge with the guanidinium group that landmarks the interactions. The R3 gating charge (Arg1605), in turn, makes van der Waals contact with the GX-936, while the R2 (Arg1602) engages the sulfonamide group of the GX-936 through its ε-nitrogen. The B- and the C-ring building blocks of the GX-936 bind alongside residues from the S2 and the S3 helices. These interactions, in particular those with Tyr1537 and Trp1538 side chains on the S2 helix, define the structural determinants of GX-936 binding to Nav1.7 VSD4-NavAb.

**Figure 3:**
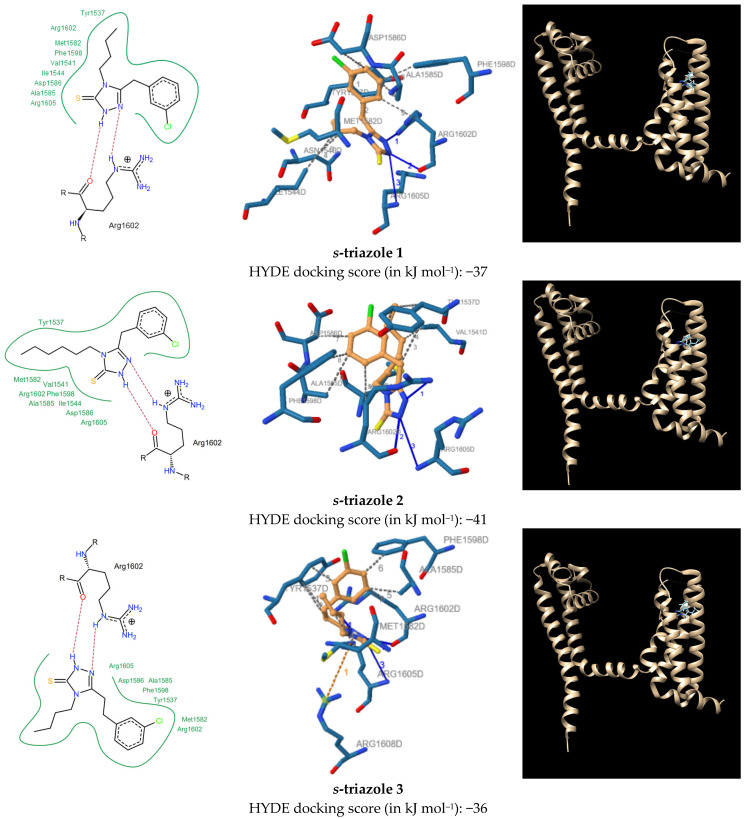

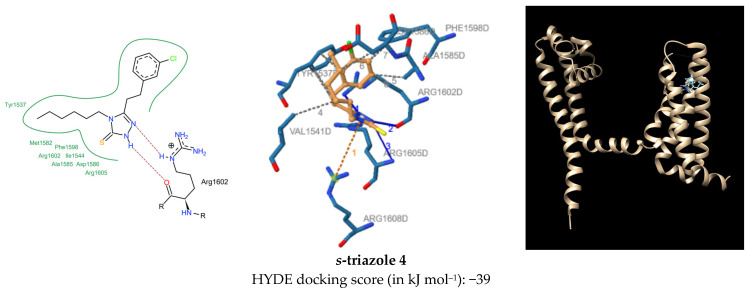
HYDE docking scores (in kJ mol^−1^) and docked conformation of the *s*-triazoles **1**–**4** in human Nav1.7-VSD4-NavAb protein (pdb id 5EK0). For details see Table 1.

In our in silico studies, the binding site was defined to include residues within a 6.5 Å radius around the native ligand (GX-936). Next, soft docking, allowing for volume overlap up to 100 Å3, was performed.

As presented in Figure 2 and Figure 3, respectively, docking of the *s*-triazoles **3** and **4** shows high correspondence to the position of the GX-936 in the X-ray structure. According to these results, the *s*-triazole ring of **3** and **4** make pi-cation interaction with the guanidinium group of the fourth gating charge residue R4 (Arg1608) on the voltage-sensing transmembrane helix (S4). As assumed based on electrophysiological studies [26], this pivotal R4 gating charge—*s*-triazole interaction can rationalize the state dependence of **3** and **4** inhibition because R4 gating charge will only be exposed to the extracellular side of the voltage sensor domain (VSD) during membrane depolarization. The R3 gating charge (Arg1605) that lines the front of the anion-binding pocket forms a hydrogen bond contact between its donor C2-amino group and the N2 nitrogen atom acceptor of the *s*-triazole ring of **3** and **4**. The ε-nitrogen of the terminal guanidinium group of the R2 gating charge (Arg1602) engages the N1 nitrogen atom of the *s*-triazole ring of **3** and **4** through a hydrogen bond interaction. The Arg1602 also shows hydrogen bond contact between its oxygen atom of carboxylic group and the NH donor group at the N2 position of *s*-triazole ring. Additionally, the Arg1602 side chain makes a hydrophobic interaction with the C5 carbonyl atom of the chlorophenyl ring **3** and **4**. According to physiological studies [27], Tyr1537 (S2) and Trp1538 (S2) side chains are recognized as major determinants of isoform-selectivity against Nav1.7. Similarly, as observed for the native antagonist GX-936, Tyr1537 forms a roof over the *s*-triazoles **3** and **4** through a number of hydrophobic interactions between its hydroxyphenyl ring and the ethylene-chlorophenyl moiety at the C5 position of the *s*-triazole. In contrast to GX-936, however, interactions between **3** and **4** with Tyr1538 side chain were not observed. Underneath the *s*-triazole **3**, in turn, Met1582 (S3) side chain forms a hydrophobic floor that supports the C4-butyl group through hydrophobic interaction. Opposite to **3** and GX-936, a direct intermolecular contact between the C4-hexyl group of the *s*-triazole **4** and Met1582 were not seen; our docking studies indicate the binding of the hexyl group to Val1541 instead. This hydrophobic interaction is particularly interesting since Val1541 (S2) side chain forms a hydrophobic floor that supports the benzonitrile and trifluoromethylphenyl rings of the GX-936 through van der Waals contacts. Among other hydrophobic interactions, Ala1585 (S3) makes a direct contact with the C5 carbon atom of the chlorophenyl ring of *s*-triazoles **3** and **4**, while the other carbon atom at the C4 position makes an interaction with the phenyl ring of Phe1598. Consistent with binding mode of GX-936, hydrophobic interaction is also observed between Asp1586 (S3) and the hexyl group of the *s*-triazole **4**. In contrast to the GX-936, however, no direct contacts of **4** as well as **3** with Gly1581 residue on the voltage-sensing S3 helix were observed. 

Thus, based on docking studies it might be concluded that designed *s*-triazoles **3** and **4** are able to inhibit Nav1.7 through a voltage-sensor trapping mechanism, similar to that depicted for the native antagonist GX-936. What is important, according to HYDE scoring function (Figure 3) the elongation of the N4 alkyl chain from the butyl to the hexyl (**1**, **2**) led to an enhanced receptor-ligand interactions, which is consistent with the experimental results [17], whereas the HYDE scores are not much different between *s*-triazoles with the methylene and the ethylene linker (**1** vs. **3** and **2** vs. **4**, respectively). Furthermore, our docking studies confirm that the binding mode of *s*-triazoles **1** and **2** is generally very similar to that of *s*-triazoles **3** and **4**, with some exceptions. Firstly, unlike the *s*-triazoles **3** and **4**, pi-cation interactions between *s*-triazoles **1** and **2** with pivotal R4 gating charge (Arg1608) are lost. Contrary to the *s*-triazoles **3** and **4**, however, for the *s*-triazoles **1** hydrophobic interactions are observed between their C4 alkyl chain and Asn1540 (S2) and Ile1544 side chains. Important to note, the residue Asn1540, strictly conserved in human Nav channels, is considered as one of the essential for binding of the anionic aryl sulfonamide warhead of native antagonist into the anion-binding pocket [25]. Furthermore, for the *s*-triazole **2**, key hydrophobic interaction between its hexyl group and Val1541 side chain is observed. Similarly, to the *s*-triazoles **3** and **4,** direct interactions between the *s*-triazoles **1** and **2** with one of the main determinants of isoform selectivity, Tyr1538 (S2), were not detected. Having this in mind, one can suppose that the *s*-triazoles **3** and **4** will have beneficial effect on VGSCs during in vitro experiments, at least as potent as that observed with their precursors **1** and **2.**

### 2.2. Chemistry

**Scheme 1:**
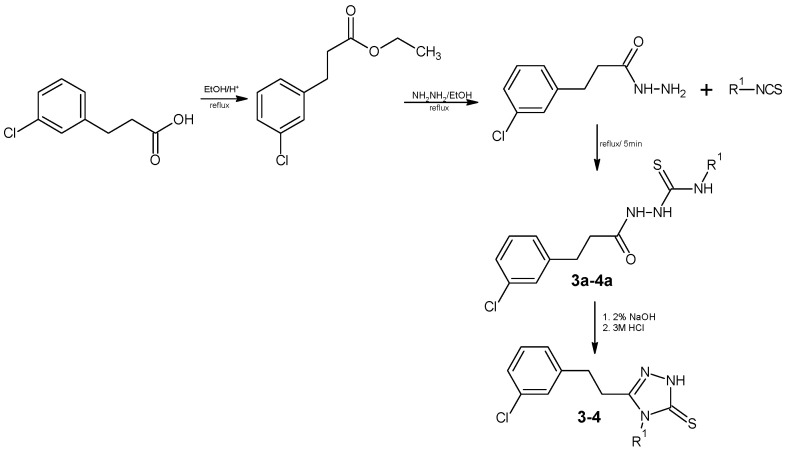
Reactions leading 4-alkyl-5-[(3-chlorophenyl)ethyl]-1,2,4-triazole-3-thione derivatives. Structures of R^1^ = *n*-butyl or *n*-hexyl.

Compounds **3** and **4** were obtained in good yields (92–94%), according to the procedure described in our previous articles [15,18,19] and depicted in Scheme 1. The structure of all obtained compounds was confirmed by spectroscopic methods: ^1^H NMR and^13^C NMR.

### 2.3. Radioligand Binding Assay for Na + Channel (Site 2) Using [^3^H]Batrachotoxin

The affinity of the designed ligands against VGSCs was examined using a radioligand binding assay, which is a laboratory technique used to study the interaction between a radiolabeled molecule (the radioligand) and a receptor or other binding site in a biological sample. [^3^H]Batrachotoxin, which was used in our research, is a radiolabeled derivative of batrachotoxin—a potent neurotoxin that binds to site 2 on the Na^+^ channel. Compounds **3** and **4** were examined in dose–response binding experiments using 8 concentrations ranging from 300 to 0.1 µM. The median inhibitory concentration (IC_50_) for compound **3** was 11.9 ± 0.4 µM and for compound **4** was 18.9 ± 0.3 µM. Both compounds possessed stronger affinity against sodium channels than carbamazepine (IC_50_ = 131 µM), an agent commonly used in the treatment of epilepsy (Table 2). Moreover, butyl derivative (**3**) displaced [^3^H]batrachotoxin from its binding site statistically significantly stronger (*p* < 0.0001) than veratridine (IC_50_ 17.8 ± 0.9 µM) (statistical analysis is presented in Table S2, see Supplementary Materials).

Contrary to our in silico calculations, the elongation of the linker between the *s*-triazole and 3-chlorophenyl group did not improve the affinity of the respective 1,2,4-triazole derivatives towards VGSCs obtained during in vitro evaluation. One can hypothesize that the differences between results obtained from in silico and in vitro experiments can result from the complex mechanisms of sodium channels inhibition. In the context of AEDs, the molecule of antagonist can bind to both the active site and another site on the target receptor or enzyme, resulting in a mixed pattern of inhibition. In such a situation, molecular docking and biological studies should be considered as two different approaches that can provide different types of information and have important differences in their scope and limitations.

### 2.4. Anticonvulsant Activity

As the sodium channel ligands are recognized as antiepileptic agents and the affinity of **3** and **4** against VGSCs was still in the beneficial range, the investigated 1,2,4-triazole derivatives were examined for their antiepileptic potential using in the vivo model of tonic–clonic seizures in mice [29]. The protective effect of compounds **3** and **4** against maximal electroshock-induced seizures (MES) was evaluated in a wide range of doses (producing 0% to 100% protection) that enabled us to calculate corresponding ED_50_ values.

Additionally, their possible acute neurotoxic effects were evaluated in the rotarod test. The obtained results (Table 2) clearly indicate that the effective doses of the tested derivatives were much lower than those causing neurotoxic effects. The protective indices (ratio of TD_50_ and ED_50_ values) for intraperitoneally administered (i.p.) compounds at various pretreatment times (i.e., 15, 30, 60, 120 min) ranged from 1.7 to even 6.2 (Table 3). Taking into account the fact that, according to the literature data, compounds with a protective index value above 5, and sometimes even 2, should be taken for further research, and if we take into account the PI values obtained by valproate, the most commonly used in pharmacotherapy of epilepsy, then it can be concluded that the compounds constitute a group of good candidates for further research [30,31]. The anticonvulsant effect of **3** and **4**, expressed as ED_50_ values at their peaks of anticonvulsant activity, was also higher than that of valproate—the most commonly used AED (*p* < 0.0001, Table S3). Higher activity was observed in the case of butyl derivative (3) (*p* < 0.0001, Table S3), which corresponds to its stronger affinity towards VGSCs. The PI value for this compound at its peak of anticonvulsant activity equaled 3.4 (Table 3). Interestingly, median toxic doses of hexyl derivative (**4**) were approximately twice as high as those calculated for compound **3** and reached the value of 710.5 mg/kg. As was to be expected on the basis of weaker VGSCs affinity of compounds containing ethylene linker (**3**, **4**), compared to their analogs with –CH_2_– linker (**1**, **2**), the anticonvulsant activity of the former derivatives was also weaker than the effect of the respective 5-(3-chlorobenzyl)-4-butyl/hexyl-1,2,4-triazole-3-thiones in the MES test (for those compounds ED_50_ values ranged from 43.2 to 102.3 mg/kg) [19]; *p* < 0.0001, Table S4.

## 3. Materials and Methods

### 3.1. Docking Methodology

Docking was performed using the HYDE scoring function [32,33,34] as implemented in the LeadIT software package. The crystal structure of human Nav1.7-VSD4-NavAb (PDB id: 5EK0) was downloaded from the Protein Data Bank (PDB). All steps of ligands and receptor preparation, including protonation states corresponding to the aqueous solution, were carried out using default settings in BioSolveIT’s LeadIT software (LeadIT version 2.3.2; BioSolveIT GmbH, Sankt Augustin, Germany, 2017). The chain D was selected, and the binding site was defined to include residues within a 6.5 Å radius around the native ligand. Soft docking (allowing for a volume overlap up to 100 Å^3^) was performed. The clash factor was set to 0.3. Other parameters were kept to default. For each ligand, the first 10 top-ranked docking poses were generated, for which HYDE analysis was performed to find out the conformation with most favorable ΔG (binding free enthalpy). The conformations with most favorable ΔG were next selected for detailed evaluation of binding site interactions. For 2D visualization, PoseView dock widget as implemented in LeadIT version 2.3.2 software was used. According to PoseView dock widget, green curves represent hydrophobic contacts of at least 3 different ligand atoms to surrounding residues. H-bond intermolecular interactions are indicated by purple dashed line. For 3D docking results visualization the PLIP (Protein Ligand Interaction Profiler) web tool was used [35]. Details of docking studies for *s*-triazoles **1**–**4** are presented in Supplementary Materials. 

The Supplementary Materials contain details of docking studies for *s*-triazoles **1**–**4.**

### 3.2. Chemistry

All reagents used for the experiments were purchased from Merck Co. (Darmstadt, Germany) and used without further purification. They had a class of the purity declared by the manufacturer. The melting points of the obtained compounds were determined with a Fisher–Johns apparatus (Fisher Scientific, Schwerte, Germany), and presented without any correction. The NMR spectra were recorded on the Bruker Avance 700 MHz apparatus (Bruker BioSpin GmbH, Ettlingen, Germany). The compounds were dissolved in dimethyl sulfoxide (DMSO-d6) before analysis. Tetramethylsilane (TMS) was used as an internal standard. Chemical shift values were given in ppm. 

#### 3.2.1. Synthesis of the Compound **3**–**4**

New 4-alkyl-5-substituted-1,2,4-triazole-3-tiones (**3**–**4**) were obtained on the basis of the procedure reported earlier [15,19]. Equimolar amounts of butyl/hexyl isothiocyanates were added to 3-(3-chlorophenyl)propanoic acid hydrazide and heated at 110 °C to form the respective thiosemicarbazide derivatives (**3a**, **4a**). The obtained compounds were washed with diethyl ether, filtered, and crystallized from anhydrous ethanol. Next, compounds **3a** and **4a** were dissolved in 2% NaOH and refluxed for 2 h. After cooling to room temperature, the solution was neutralized with 3M HCl. The corresponding 1,2,4-triazole derivatives (**3**–**4**) were then filtered off under reduced pressure, dried and, crystallized from anhydrous ethanol.

##### 4-Butyl-1-[(3-chlorophenyl)propionyl]thiosemicarbazide (**3a**)

Yield: 78%, m.p. 108–110 °C, ^1^H NMR (700 MHz): 0.87 (t, 3H, CH_3_, *J* = 7.0 Hz), 1.30 (sext., 2H, CH_2_, *J* = 7.0 Hz), 1.53 (quint., 2H, CH_2_, *J* = 7.1 Hz), 2.56 (t, 2H, CH_2_, *J* = 7.2 Hz), 2.95 (t, 2H, CH_2_, *J* = 7.3 Hz), 3.43–3.48 (m, 2H, CH_2_), 7.11–7.42 (m, 4H, ArH), 7.97 (s, 1H, NH), 9.06 (s, 1H, NH), 9.57 (s, 1H, NH). ^13^C NMR (175 MHz): 13.85, 20.42, 31.12, 31.38, 33.25, 44.08, 127.80, 128.38, 129.29, 129.71, 134.50, 136.05, 171.31, 181.90. Anal. C_14_H_20_ClN_3_OS (C, H, N).

##### 1-[(3-Chlorophenyl)propionyl]-4-hexylthiosemicarbazide (**4a**)

Yield: 74%, m.p. 110–112 °C, ^1^H NMR (700 MHz): 0.85 (t, 3H, CH_3_, *J* = 7.0 Hz), 1.23–1.32 (m, 6H, 3CH_2_), 1.59 (quint., 2H, CH_2_, *J* = 6.9 Hz), 2.61 (t, 2H, CH_2_, *J* = 7.2 Hz), 2.99 (t, 2H, CH_2_, *J* = 7.2 Hz), 3.41–3.46 (m, 2H, CH_2_), 7.09–7.48 (m, 4H, ArH), 7.80 (s, 1H, NH), 8.42 (s, 1H, NH), 9.48 (s, 1H, NH). ^13^C NMR (175 MHz): 14.10, 22.62, 26.55, 29.96, 31.43, 31.72, 33.58, 43.18, 124.96, 127.48, 129.56, 131.21, 133.74, 136.28, 170.37, 181.67. Anal. C_16_H_24_ClN_3_OS (C, H, N).

##### 4-Butyl-5-[2-(3-chlorophenyl)ethyl]-2,4-dihydro-3*H*-1,2,4-triazole-3-thione (**3**)

Yield: 92%, m.p.102–104 °C,^1^H NMR (700 MHz): 0.89 (t, 3H, CH_3_, J = 7.3 Hz), 1.28 (quint, 2H, CH_2_, J = 7.4 Hz), 2.98–3.03 (m, 4H, 2CH_2_), 3.88 (t, 2H, CH_2_, J = 7.5 Hz), 7.24–7.42 (m, 4H, ArH), 13.50 (s, 1H, NH). ^13^C NMR (175 MHz): 14.02, 19.80, 26.35, 30.24, 31.06, 43.09, 126.66, 127.75, 128.85, 130.58, 133.38, 143.43, 151.88, 166.83. Anal. C_14_H_16_ClN_3_S (C, H, N).

##### 5-[2-(3-Chlorophenyl)ethyl]-4-hexyl-2,4-dihydro-3*H*-1,2,4-triazole-3-thione (**4**)

Yield: 94%, m.p. 84–86 °C, ^1^H NMR (700 MHz): 0,72 (t, 3H, CH_3_, *J* = 6.5 Hz), 1.04–1.21 (m, 6H, 3CH_2_), 1.34–1.54 (m, 2H, CH_2_), 2.83–2.93 (m, 4H, 2CH_2_), 3.75 (t, 2H, CH_2_, *J* = 7.7 Hz), 7.08–7.31 (m, 4H, ArH), 13.39 (s, 1H, NH). ^13^C NMR (175 MHz): 13.83, 21.94, 25.62, 25.87, 27.54, 30.63, 30.71, 42.71, 126.20, 127.27, 128.35, 130.07, 132.93, 142.93, 151.39, 166.27. Anal.C_16_H_22_ClN_3_S (C, H, N).

### 3.3. Radioligand Binding Assay for Na + Channel (Site 2) Using [^3^H]Batrachotoxin

The radioligand binding assay was performed according to the method previously described by Callaway et al. [36], with slight modifications [18]. Rat cerebral cortex was used to obtain membrane fraction. The investigated compounds were initially tested at a concentration of 100 µM. In order to calculate the respective IC_50_ values, the compounds were tested in concentrations ranging from 0.1 to 300 µM. Dose–response curves and median inhibitory concentrations were obtained using GraphPad Prism 7 (GraphPad Software, San Diego, CA, USA).

### 3.4. Animal Experimentations

All animal experiments were performed in accordance with EU Directive 2010/63/EU for animal experiments and complied with the ARRIVE guidelines. All the procedures were also approved by the Local Ethics Committee (Lublin, Poland).

#### 3.4.1. MES Test

Anticonvulsant activity of the investigated compounds was determined according to the same procedure as described in [11,15,18]. Adult albino mice weighing 20–25 g were used in the experiment. The animals were randomly divided into groups of 8 mice and kept in colony cages with free access to water and food. Test compounds were administered intraperitoneally, suspended in a 1% solution of Tween 80 in distilled water in a volume of 5 mL/kg. Electroconvulsions were produced with alternating current (0.2 s stimulus duration; 500 V, 50 Hz, 25 mA constant current) delivered through ear clip electrodes by a type 221 rodent shock generator (Hugo Sachs Elektronik, Freiburg, Germany). Absence of tonic extension of the hind limb was assumed as the criterion of anticonvulsant activity. ED_50_ values were calculated using Litchfield and Wilcoxon’s log-probit method.

#### 3.4.2. Rotarod Test

Acute neurotoxicity of the compounds obtained after i.p. administration was tested in mice using the rotarod test according to the methodology described earlier [37]. The neurotoxicity of the tested compounds is presented as median toxic doses (TD_50_) that impair motor coordination in 50% of the mice challenged with the rotarod test.

### 3.5. Statistical Analysis

Differences between the median inhibitory concentrations (IC_50_) or median effective doses (ED_50_) of the investigated compounds were analyzed using one-way ANOVA followed by Tukey’s multiple comparisons test. The results are presented in Tables S2–S4.

## 4. Conclusions

Although our previous results and currently performed docking simulations justified the higher affinity of 5-[(3-chlorophenyl)ethyl]-4-butyl/hexyl-1,2,4-triazole-3-thiones towards VGSCs compared to the respective compounds with only a methylene linker between the 1,2,4-triazole ring and halogenophenyl moiety, the in vitro radioligand binding assay did not confirm these assumptions. While the investigated triazole-based ligands (**3**, **4**) still acted as VGSC ligands, their affinities were lower than those of the respective analogs containing shorter alkyl linkers. One can hypothesize that the differences between results obtained from in silico and in vitro experiments can result from the complex mechanisms of sodium channels inhibition, in which the ligands can bind to both the active site and another site on the target receptor, resulting in a mixed pattern of inhibition. Importantly, the anticonvulsant activity effect of **3** and **4**, at the peak of their anticonvulsant action, was still higher than that of valproate—the most commonly used AED. Median effective doses (ED_50_) of the compounds examined in the MES test reached 96.6 ±14.8 mg/kg, while their median toxic doses (TD_50_), obtained in the rotarod test, were even as high as 710.5 ± 47.4 mg/kg.

## Figures and Tables

**Table 1:** Noncovalent interactions between the *s*-triazoles **1**–**4** with human Nav1.7-VSD4-NavAb protein (pdb id 5EK0).

*s*-Triazole	Hydrophobic Interactions and Distance ······	H-Bond Interactions —	H-A Distance	D-A Distance	pi-Cation Interactions ······
**1**	Tyr1537 (3.06 Å, 3.86 Å), Asn1540 (3.44 Å), Ile1544 (3.81 Å), Met1582 (3.45 Å), Ala1585 (3.25 Å), Asp1586 (3.85 Å), Phe1598 (2.97 Å), Arg1602 (3.49 Å)	Arg1602 Arg1602 Arg1605	3.01 Å 1.81 Å 3.44 Å	3.63 Å 2.71 Å 4.02 Å	—
**2**	Tyr1537 (3.68 Å, 2.90 Å, 3.65 Å), Val1541 (3.31 Å), Met1582 (3.60 Å), Ala1585 (3.44 Å), Asp1586 (3.54 Å), Phe1598 (3.73 Å), Arg1602 (3.92 Å)	Arg1602 Arg1602 Arg1605	2.90 1.82 3.49	3.53 2.69 4.09	—
**3**	Tyr1537 (3.39 Å, 3.51 Å, 3.02 Å), Met1582 (3.85 Å), Ala1585 (3.11 Å), Phe1598 (2.81 Å), Arg1602 (3.14 Å)	Arg1602 Arg1602 Arg1605	2.68 1.94 3.54	3.40 2.90 4.07	Arg1608 (5.83 Å)
**4**	Tyr1537 (3.60 Å, 3.66 Å, 3.19 Å), Val1541 (3.99 Å), Ala1585 (3.12 Å), Asp1586 (3.91 Å), Phe1598 (3.15 Å), Arg1602 (3.36 Å)	Arg1602 Arg1602 Arg1605	2.71 1.88 3.46	3.44 2.85 3.99	Arg1608 (5.73 Å)

**Table 2:** In vitro measurement of the ability of the investigated 1,2,4-triazole derivatives to interact with the batrachotoxin-binding sites of VGSCs.

Compound Number	Compound Structures	Na^+^ Channel (Site 2) Affinity IC_50_ [µM] ± SEM	Dose—Response Binding Curves
**3**	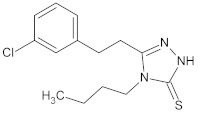	11.9 ± 0.4	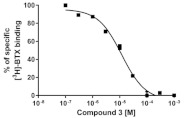
**4**	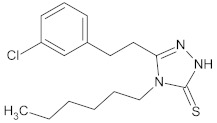	18.9 ± 0.3	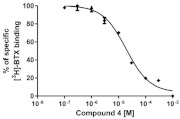
	Veratridine	17.8 ± 0.9	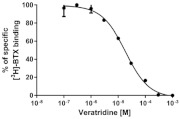
	Carbamazepine	131 ^1^	

^1^ Data obtained from [28].

**Table 3:** Anticonvulsant activity (MES test) and neurotoxicity (rotarod test) of the investigated 1,2,4-triazole-3-thione derivatives.

Compound Number	Compound	Anticonvulsant Activity		Neurotoxicity	
		Pretreatment Time (min)	ED_50_ (mg/kg) ± SEM	TD_50_ (mg/kg) ± SEM	PI (TD_50_/ED_50_)
**3**	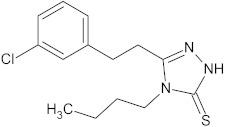	15	138.7 ± 31.4	329.5 ± 30.0	2.4
		30	96.6 ± 14.8	329.5 ± 30.0	3.4
		60	148.3 ± 17.1	337.7 ± 26.3	2.3
		120	194.9 ± 18.9	325.9 ± 23.1	1.7
**4**	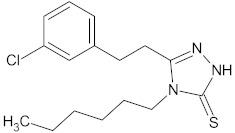	15	115.4 ± 7.6	710.5 ± 47.4	6.2
		30	129.0 ± 21.1	685.4 ± 49.0	5.3
		60	214.9 ± 21.1	698.4 ± 46.2	3.2
		120	254.8 ± 22.5	478.9 ± 49.6	1.9
	Valproate ^1^	15	189.0 ± 17.3	363.3 ± 14.2	1.9
		30	216.9 ± 9.4	372.9 ± 16.9	1.7
		60	218.4 ± 18.9	417.3 ± 9.5	1.9
		120	246.6 ± 21.4	512.3 ± 30.2	2.1

^1^ Data obtained from [19].

## Data Availability

The data presented in this study are available on request from the corresponding author.

## Supplementary Materials

The following supporting information can be downloaded at: https://www.mdpi.com/article/10.3390/molecules28135287/s1, Table S1. Docking results of the *s*-triazoles **1**–**4**– in human Nav1.7-VSD4-NavAb protein (pdb id 5EK0). Table S2. Statistical evaluation of differences between means of IC_50_ values reflecting VGSC affinity. Table S3. Statistical evaluation of differences between means of ED_50_ values of compounds **3**, **4** and valproate measured at the peak of their anticonvulsant activity. Table S4. Statistical analysis of differences between ED_50_ values showing anticonvulsant activity of compounds containing metylene linker (**1**, **2**) and ethylene linker (**3**, **4**).

## Sample Availability

Samples of the compounds are available from the authors.
