# Supplementary material for: Effect of Linker Elongation on the VGSC Affinity and Anticonvulsant Activity among 4-Alkyl-5-aryl-1,2,4-triazole-3-thione Derivatives
Source: Molecules. 2023 Jul 7;28(13):5287. doi: 10.3390/molecules28135287 (PMC10343781; doi:10.3390/molecules28135287)
Supplement: Supplementary file 1 [file molecules-28-05287-s001.zip › molecules-2395961-supplementary.pdf]

SUPPORTING INFORMATION

Table S1. Docking results of the *s*-triazoles **1-4** in human Nav1.7-VSD4-NavAb protein (pdb id 5EK0)

s-triazole	Posename #	docking score	Hyde (ΔG) in kJ/mol	PoseView
**1**	**1**	-13.4	-37	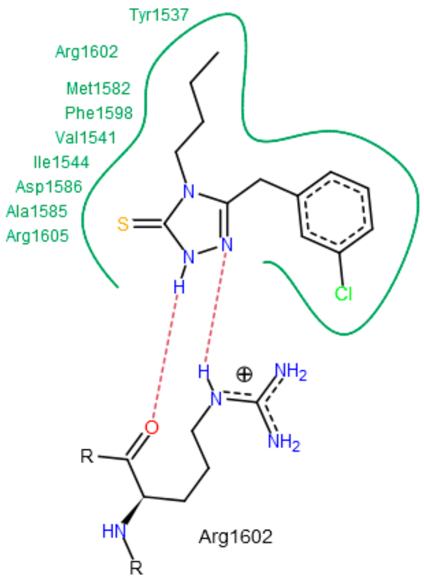

<table>
<tr><td></td><td>2</td><td>-13.0</td><td>-36</td><td>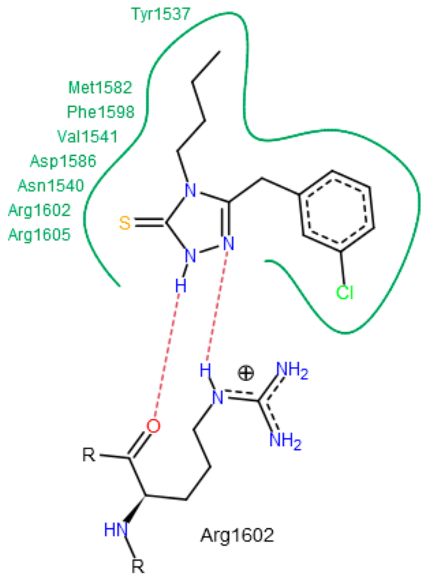
</td></tr>
<tr><td></td><td>3</td><td>-12.7</td><td>-37</td><td>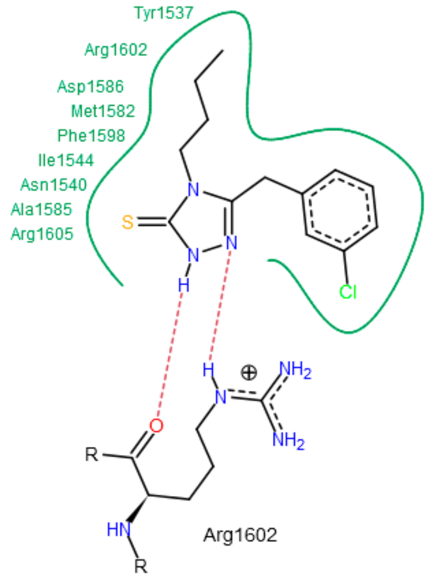
</td></tr>
</table>

	**4**	-12.4	-25	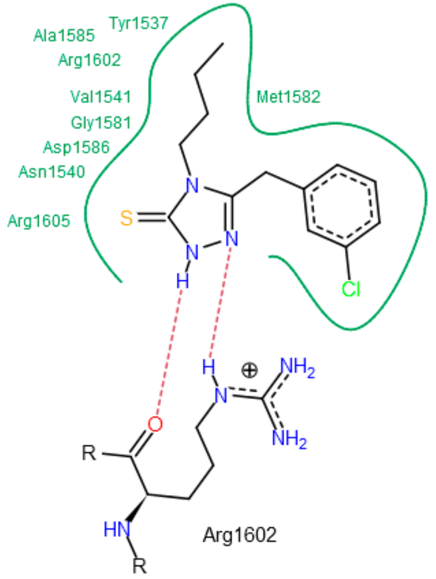
	**5**	-12.0	-28	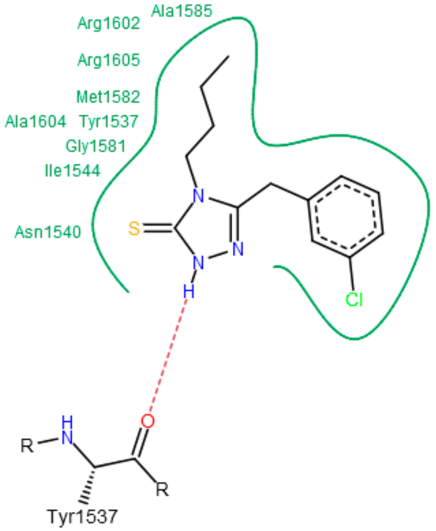

	**6**	-12.0	-28	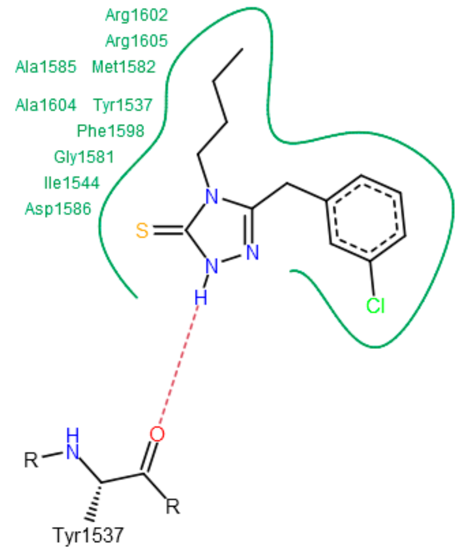
	**7**	-11.8	-23	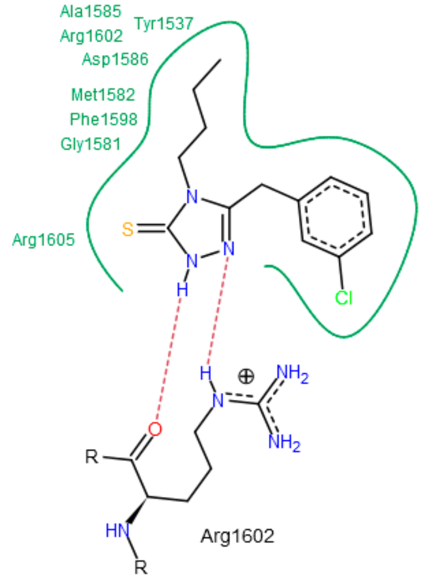

	**8**	-11.8	-10	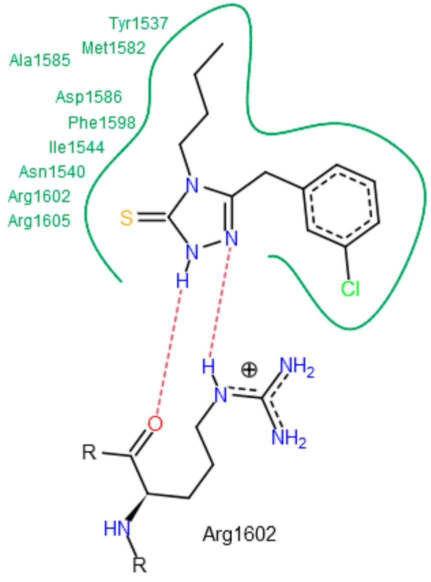
	**9**	-11.8	-28	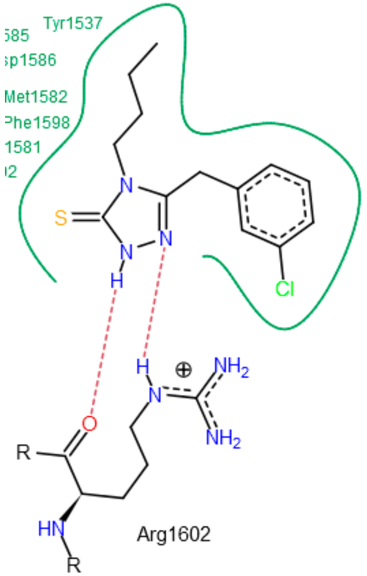

	**10**	-11.7	-31	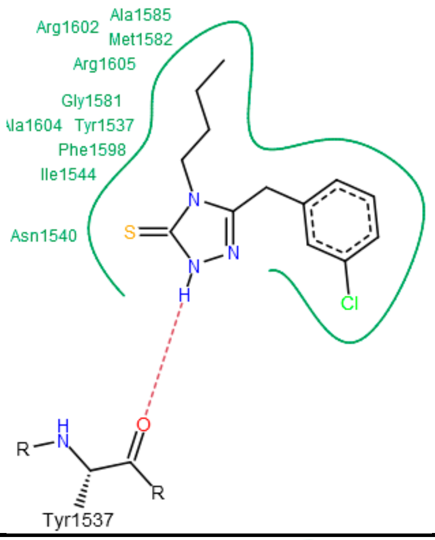
**2**	**1**	-12.1	-40	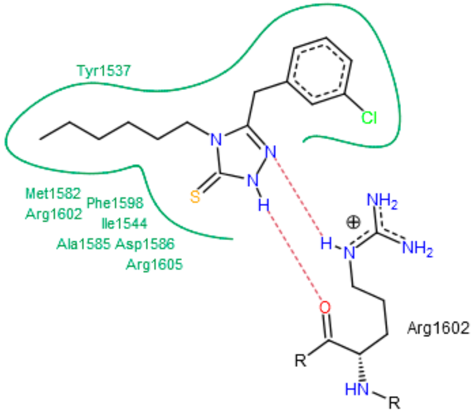

	**2**	-12.0	-6	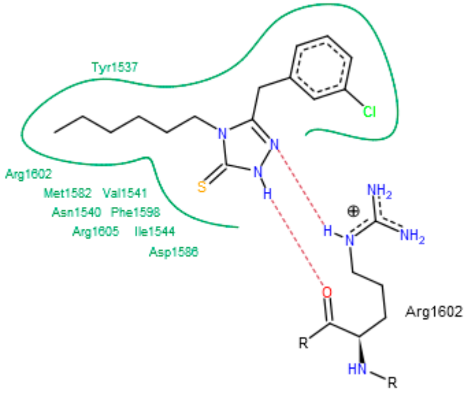
	**3**	-11.3	-41	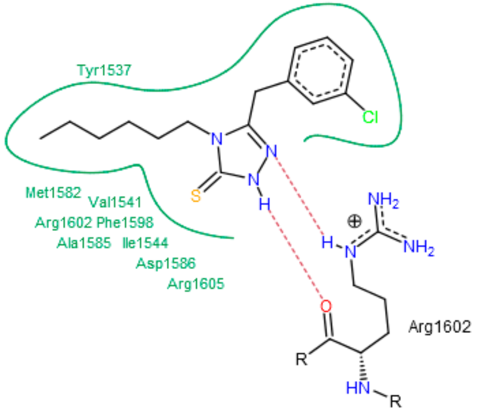

	**4**	-11.0	-14	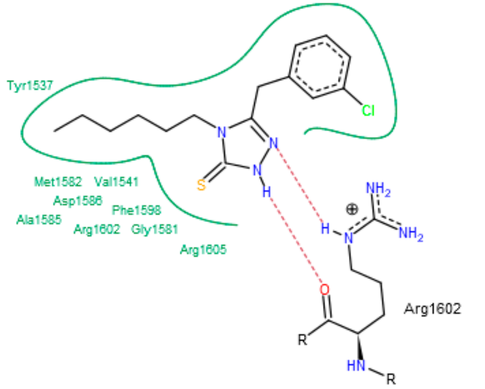
	**5**	-11.0	-26	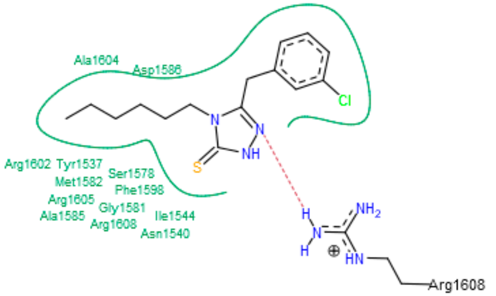
	**6**	-10.9	-8	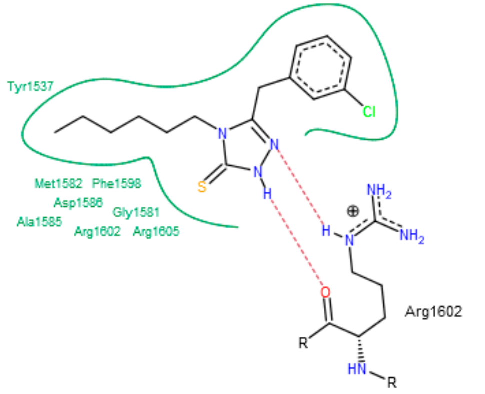

	**7**	-10.8	-17	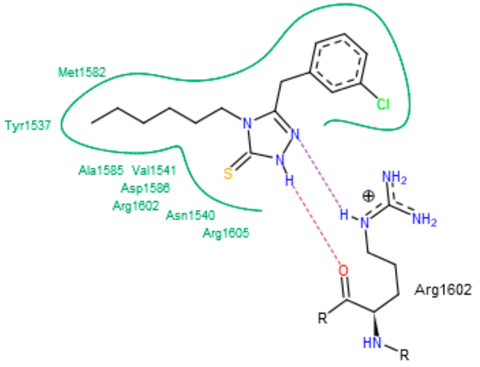
	**8**	-10.7	-32	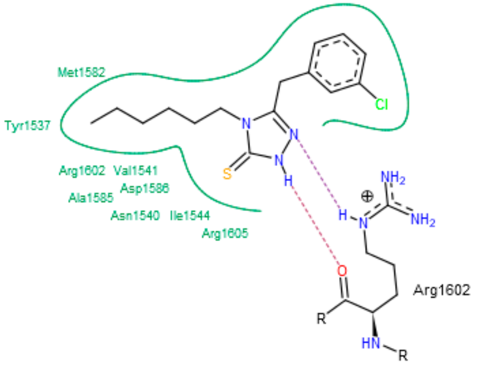
	**9**	-10.6	-8	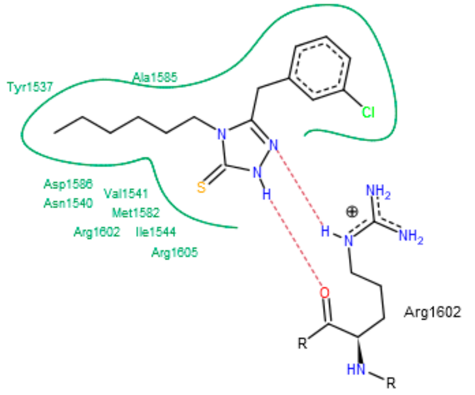

	**10**	-10.4	-29	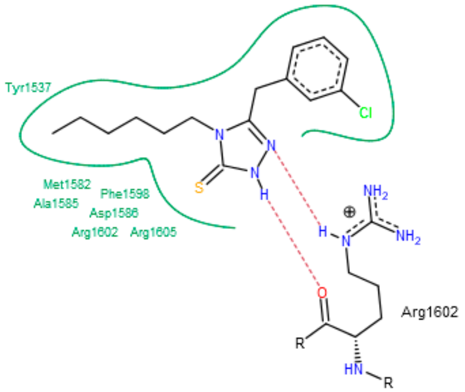
**3**	**1**	-13.2	-13	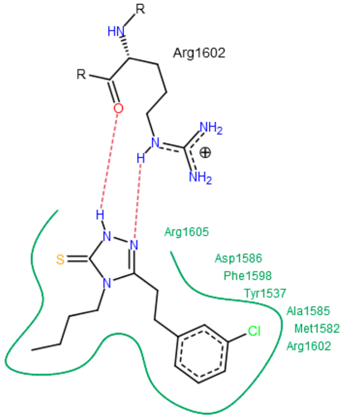

	**2**	-12.4	-21	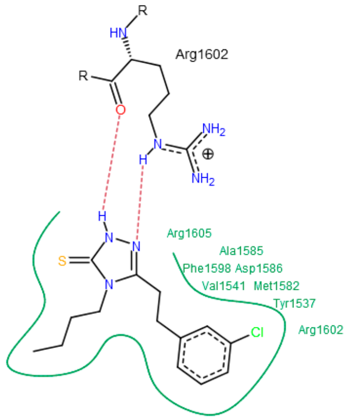
	**3**	-11.4	-11.0	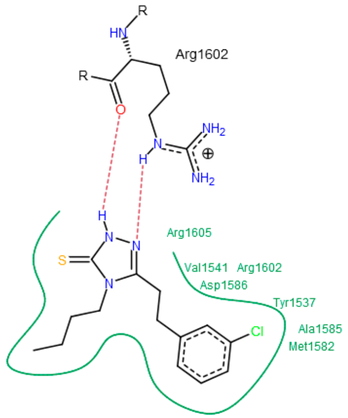

	**4**	-11.0	-34	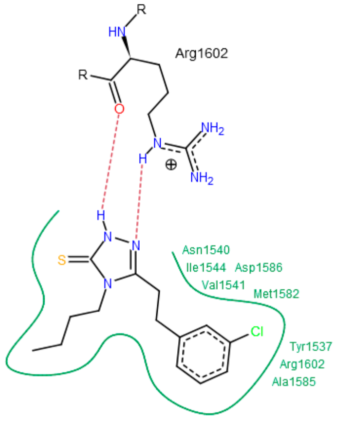
	**5**	-10.8	-24	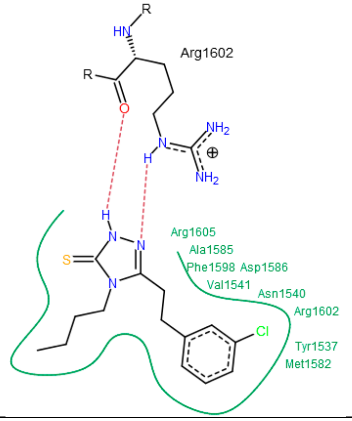

	**6**	-10.7	-36	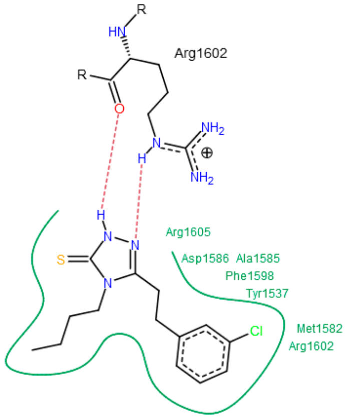
	**7**	-10.7	-34	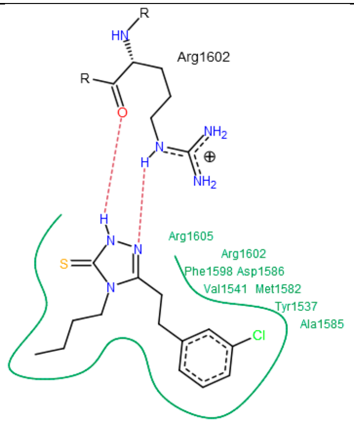

	**8**	-10.6	-34	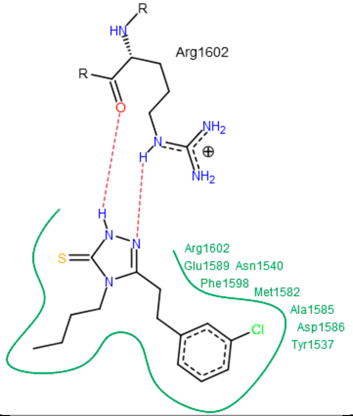
	**9**	-10.6	-17	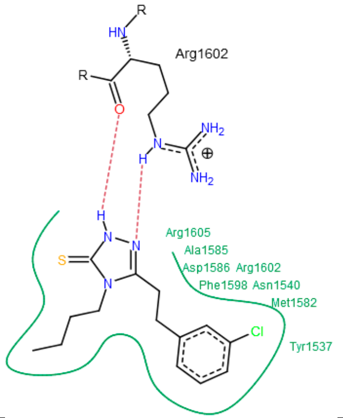

	**10**	-10.2	-27	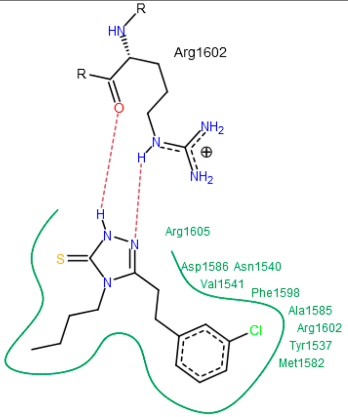
**4**	**1**	-11.6	-25	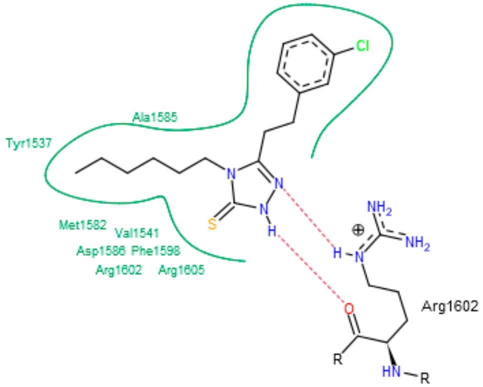
	**2**	-11.6	-22	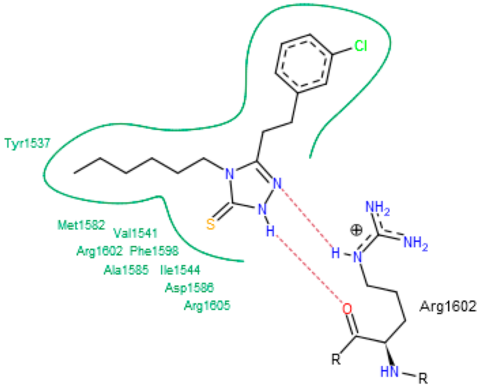

	**3**	-11.5	-39	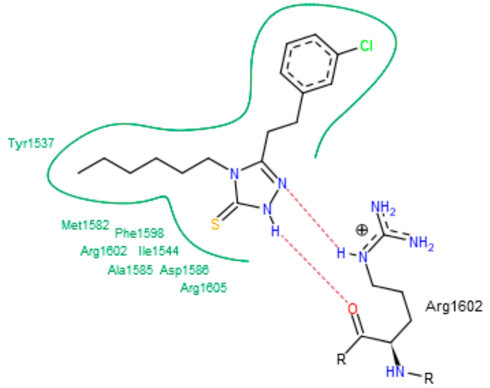
	**4**	-11.4	-30	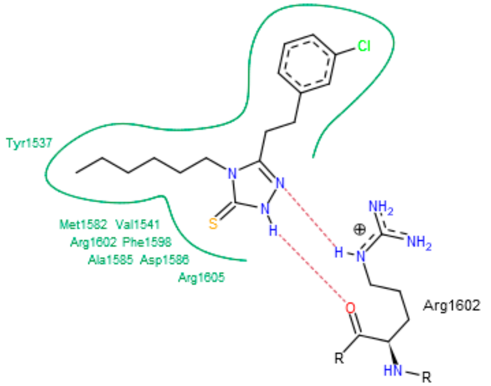
	**5**	-11.3	-14	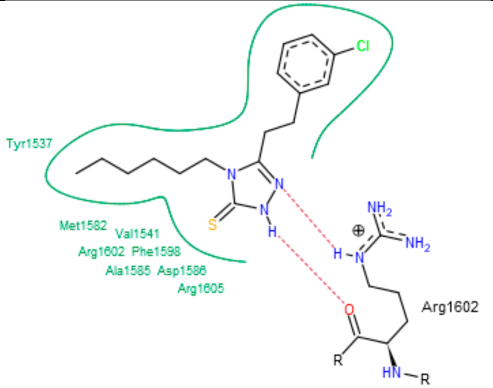

	**6**	-10.9	-16	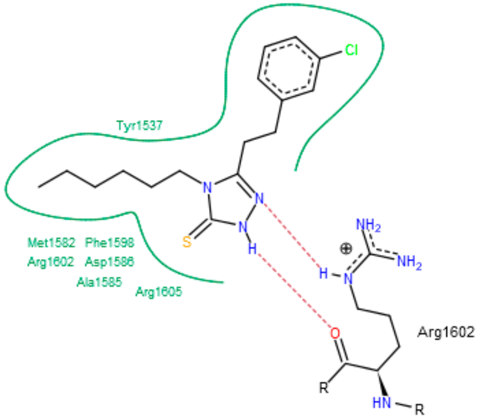
	**7**	-10.6	-31	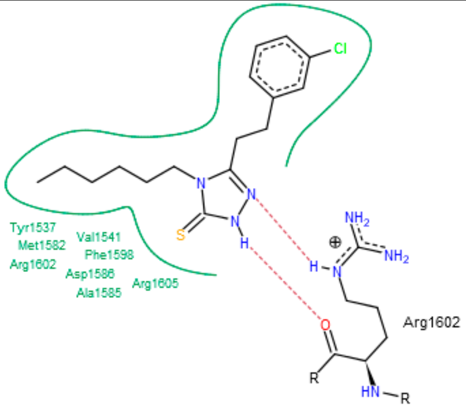

	**8**	-10.5	-14	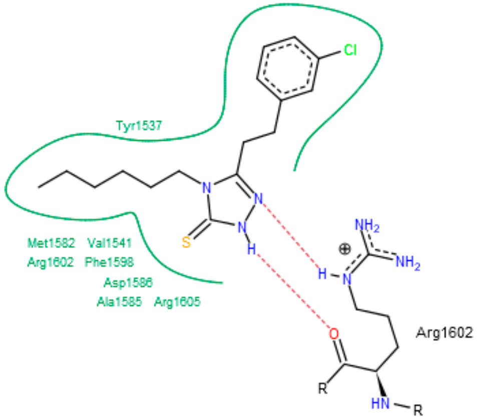
	**9**	-10.5	-12	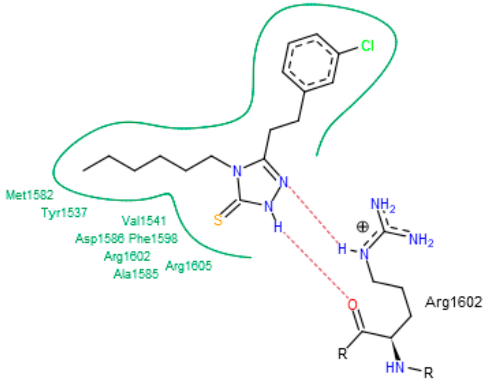
	**10**	-10.3	-10	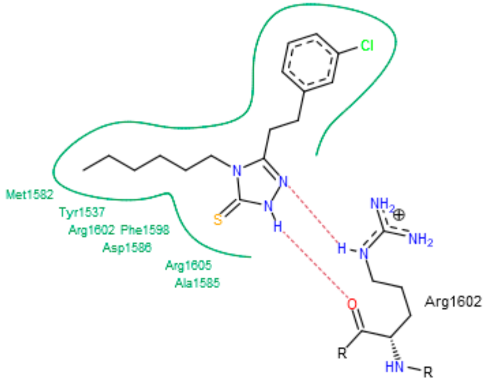

Table S2. Statistical evaluation of differences between means of IC_{50} values reflecting VGSC affinity.

Tukey's multiple comparisons test	Mean diff.	95.00% CI of diff.	Significant?	Summary	Adjusted P value
1 vs. 2	9.63	9.152 to 10.11	Yes	****	<0.0001
1 vs. 3	3.9	3.422 to 4.378	Yes	****	<0.0001
1 vs. 4	-3.1	-3.578 to -2.622	Yes	****	<0.0001
1 vs. veratridine	-2	-2.478 to -1.522	Yes	****	<0.0001
2 vs. 3	-5.73	-6.208 to -5.252	Yes	****	<0.0001
2 vs. 4	-12.73	-13.21 to -12.25	Yes	****	<0.0001
2 vs. veratridine	-11.63	-12.11 to -11.15	Yes	****	<0.0001
3 vs. 4	-7	-7.478 to -6.522	Yes	****	<0.0001
3 vs. veratridine	-5.9	-6.378 to -5.422	Yes	****	<0.0001
4 vs. veratridine	1.1	0.622 to 1.578	Yes	****	<0.0001

Table S3. Statistical evaluation of differences between means of ED_{50} values of compounds **3**, **4** and valproate measured at the peak of their anticonvulsant activity.

Tukey's multiple comparisons test	Mean diff.	95.00% CI of diff.	Significant?	Summary	Adjusted P value
ED_{50} of 3 vs ED_{50} of valproate	-92.4	-102 to -82.82	Yes	****	<0.0001
ED_{50} of 4 vs ED_{50} of valproate	-73.6	-83.18 to -64.02	Yes	****	<0.0001
ED_{50} of 3 vs ED_{50} of 4	-18.8	-28.38 to -9.218	Yes	****	<0.0001

Table S4. Statistical analysis of differences between ED_{50} values showing anticonvulsant activity of compounds containing metylene linker (**1**, **2**) and ethylene linker (**3**, **4**)

Tukey's multiple comparisons test	Mean diff.	95.00% CI of diff.	Significant?	Summary	Adjusted P value
ED_{50} of 1 vs ED_{50} of 3 (*butyl derivatives*)	-53.4	-61.25 to -45.55	Yes	****	<0.0001
ED_{50} of 2 vs ED_{50} of 4 (*hexyl derivatives*)	-43.3	-51.15 to -35.45	Yes	****	<0.0001
